# A retrospective observational study of serum uric acid and in-hospital mortality in acute type A aortic dissection

**DOI:** 10.1038/s41598-022-16704-3

**Published:** 2022-07-19

**Authors:** Guifang Yang, Xiangping Chai, Ning Ding, Donghua Yang, Qiong Ding

**Affiliations:** 1 Department of Emergency Medicine, The Second Xiangya Hospital, Central South University, Changsha, China; 2 Trauma Center of Hunan Province, Changsha, China; 3 Department of Nursing, The Affiliated Changsha Central Hospital, Hengyang Medical School, University of South China, NO. 161 Shaoshan South Road, Changsha, 410004 Hunan China

**Keywords:** Cardiology, Risk factors

## Abstract

There is currently insufficient evidence of correlation between on-admission serum uric acid and in-hospital mortality of patients with acute type A aortic dissection. Thus, this study analysed the relation between serum uric acid and in-hospital deaths in patients with acute type A aortic dissection. A total of 1048 patients with acute type A aortic dissection participated in this study between January 2010 and December 2018. The independent variable was on-admission serum uric acid, whilst the dependent variable was in-hospital deaths. The covariates of the study included patient age, gender, body mass index, smoking status, hypertension, diabetes, Marfan syndrome, bicuspid aortic valve, chronic renal insufficiency, stroke, atherosclerosis, time to presentation, systolic blood pressure, diastolic blood pressure, aortic diameter, aortic regurgitation, abdominal vessel involvement, arch vessel involvement, ejection fraction value, laboratory parameters, symptom, coronary malperfusion, mesenteric malperfusion, cerebral malperfusion, hypotension/shock, cardiac tamponade and operation status. The mean age of the sample was 50.17 ± 11.47 years, with approximately 24.24% of the participants being female. After analysis, it was found that the admission serum uric acid of patients with acute type A aortic dissection was positively correlated with in-hospital death (OR = 1.04, 95% CI 1.02–1.06). Subsequently, a non-linear relationship was determined between admission serum uric acid (point 260 µmol/L) and in-hospital mortality for patients with acute type A aortic dissection. The effect sizes and confidence intervals of the right (serum uric acid > 260 µmol/L) and left (serum uric acid ≤ 260 µmol/L) aspects of the inflection point were 1.04 (1.02–1.05) and 1.00 (0.99–1.02), respectively. Furthermore, subgroup analysis indicated a stable relationship between serum uric acid and in-hospital mortality, whilst an insignificant difference was found for the interactions between different subgroups. Overall, a non-linear correlation was determined between admission serum uric acid and in-hospital mortality of patients with acute type A aortic dissection. When serum uric acid > 260 µmol/L, it showed a positive correlation with in-hospital mortality.

## Introduction

Acute type A aortic dissection (ATAAD) is a serious medical condition linked with high morbidity and mortality^1^. The data on the incidence of ATAAD vary substantially. According to previous research, the incidence of ATAAD was 11.9 cases in 100,000 patients per year for the whole Berlin-Brandenburg region and 5.93–24.92 cases/100,000 inhabitants/year among different emergency department^2,3^. Despite the potentially life-threatening consequences of this issue, there is currently a lack of effective indicators to assess the prognosis of aortic dissection^4^. A lot of studies have attempted to identify risk factors for in-hospital mortality in ATAAD patients like pulse deficit, left ventricular systolic dysfunction, renal dysfunction, and so on^5,6^. In addition, Augoustides et al.^7^ established the Penn classification to enable stratification of ATAAD patients by operative mortality risk. However, studies on the relationship between uric acid levels at admission and ATAAD prognosis are lacking. Uric acid is a heterocyclic organic compound that becomes a final product of purine metabolism in humans^8^. Several research findings have indicated an association between elevated levels of UA and cardiovascular disease (CVD)^9–11^. One particular study included a 23-year follow-up period, whereby this link between serum UA levels and cardiovascular outcomes was first reported in the general population based on analysis of established cardiovascular risk factors^12^. Patients diagnosed with aortic diseases, such as aortic aneurysm rupture and aortic dissection, generally have higher UA levels than individuals without aortic diseases, yet it remains undetermined whether serum UA influences ATAAD-related mortality^13,14^. Therefore, this study aims to investigate serum UA levels following patient admission and in-hospital mortality among patients with ATAAD, following adjustment for confounding variables.

## Methods and participants

### Study design

The independent variable of this study was baseline admission UA of participants, whilst in-hospital mortality was selected as the dependent variable. The study followed a retrospective, observational approach.

### Study population

Data was collected from consecutive patients with ATAAD on a non-selective basis at the Second Xiangya Hospital of Central South University, Hunan, China. Permission was granted to access the electronic hospital medical record system to obtain the required data. The sample population comprised 1048 inpatients who had received medical treatment at the hospital between January 2010 and December 2018. ATAAD diagnosis was defined as a dissection involving the ascending aorta whereby presentation had occurred within 14 days of symptom onset. A confirmatory diagnosis was secured through standard imagine techniques, primarily computed tomography or magnetic resonance imaging. The inclusion criterion was ATAAD diagnosis; the exclusion criteria included incomplete UA values, the detection of intramural haematoma, and symptoms lasting for more than 14 days.

### Ethics declarations

This study was performed in accordance with the Declaration of Helsinki. Patient identity remained anonymous, and the requirement for informed consent was deferred due to the observational nature of the study. Ethical approval was obtained prior to commencement of the study from the Ethics Committee of the Second Xiangya Hospital, Central South University (Changsha, China, No. 2020-514). All methods were performed in accordance with the guidelines and regulations.

### Variables

In this study, in-hospital mortality refers to all-cause deaths during the period of admission. The covariates were patient demographics, biochemical profiles, imaging examinations, and treatment factors that could potentially influence admission serum UA or in-hospital mortality. Based on this list, the fully adjusted model involved the following continuous variables at baseline: patient age, body mass index (BMI), time to presentation, systolic blood pressure (SBP), diastolic blood pressure (DBP), aortic diameter (the diameter of the aortic root), ejection fraction (EF), triglyceride (TG), total cholesterol (TC), high-density lipoprotein (HDL), low-density lipoprotein (LDL), creatinine (Cr), blood urea nitrogen (BUN), estimated glomerular filtration rate (eGFR), D-dimer, fibrinogen and fibrin degradation products (FDP), and C-reactive protein (CRP). In regard to the categorical variables of the model: gender, smoker/nonsmoker status, diabetes, hypertension, Marfan syndrome, bicuspid aortic valve, stroke, atherosclerosis (previous coronary atherosclerotic heart disease (CHD) and/or carotid plaque), aortic regurgitation (Grade I-IV all included), chronic renal insufficiency (CRI, pre-existing chronic renal insufficiency (Cr more than 133 umol/L) or requiring maintenance hemodialysis), abdominal vessel involvement, arch vessel involvement, symptom, coronary malperfusion, mesenteric malperfusion, cerebral malperfusion, hypotension/shock, cardiac tamponade and operation status.

### Addressing missing data

Issues regarding statistical power and bias in relation to missing data were approached through the conduction of multiple multivariable imputations. The MICE software package in R facilitates the creation of required imputation datasets and Rubin’s rules were followed^15^; subsequently, significant differences were not found between the generated data and the raw data following sensitivity analysis.

### Statistical analysis

The categorical variables are presented as percentages, whilst the continuous variables are depicted as the mean ± SD or the lower and upper quartile values (25th, 75th). Statistical testing involved the Kruskal Wallis H test, analysis of variance (ANOVA), or chi-squared test; this enabled the analysis of normally distributed data, including the analysis of discrepancies between different admission serum UA groups (tertile). Correlation between admission serum UA and in-hospital mortality was explored by univariate and multivariate regression (linear) models. In addition, the fitting of an additive-generalised model and the penalised spline method was implemented to target nonlinearity in admission UA values and in-hospital mortality. Determination of non-linearity subsequently resulted in the utilisation of a recursive algorithm in order to calculate the point of inflection; this was followed by the construction of a linear two-piece regression. In regard to the likelihood log-ratio test, the best fit model was assessed against the p values. Furthermore, subgroup analyses were achieved using a stratified linear regression model. Following Kaplan–Meier analysis and parallels with the test on log-rank, survival curves were created. EmpowerStats (X&Y Inc Solutions, Boston, MA) and R were used to complete statistical analyses. Statistical significance was confirmed when *p* =  < 0.05 (two-sided).

### Ethics approval and consent to participate

As a result of its retrospective, informed consent was waived by the Ethics Committee of the Second Xiangya Hospital, Central South University (Changsha, China).

## Results

### Baselines characteristics of study participants

**Figure 1:**
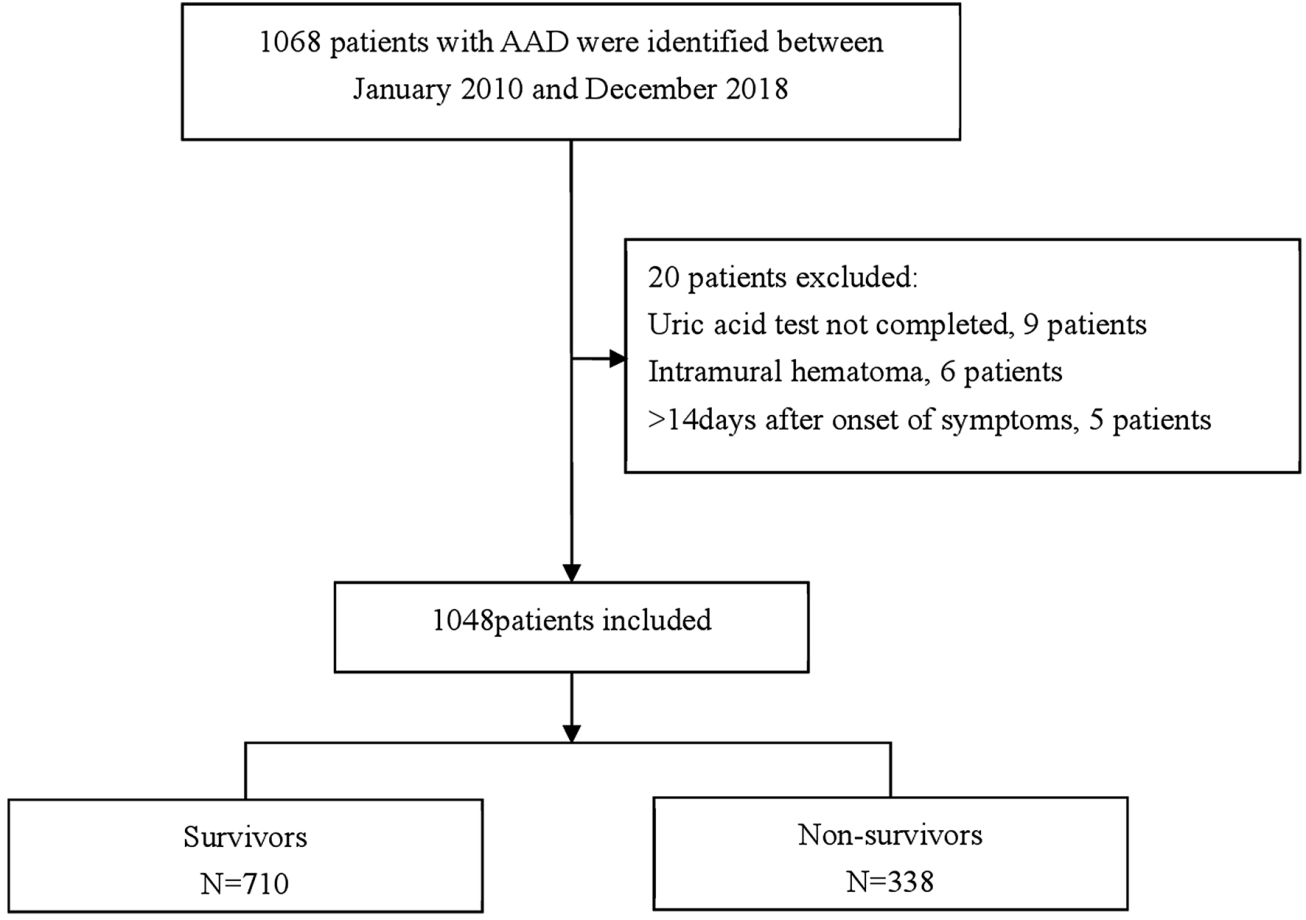
Patient enrollment process.

Adherences to the inclusion and exclusion criteria resulted in the attainment of 1,048 study participants (Fig. 1). Table 1 shows the baseline tertile admission serum UA values of these patients. The average participant age was 50.17 ± 11.47 years; also, 24.24% of the sample were female participants. Participants from the uppermost group of admission serum UA (T3) presented with relatively higher baselines values of BMI, CRI, Cr, BUN, TG, D-dimer, FDP, coronary malperfusion and mortality. This was also noted for gender (female) and operation status in T1 groups. Statistical significance was not found for age, smoker/nonsmoker status, diabetes, hypertension, Marfan syndrome, stroke, bicuspid aortic valve, atherosclerosis, time to presentation, SBP, DPB, aortic diameter, aortic regurgitation, abdominal vessel involvement, arch vessel involvement, symptom, mesenteric malperfusion, cerebral malperfusion, hypotension/shock, cardiac tamponade, TC, HDL, and LDL among the admission serum UA groups (*p* =  > 0.05).

**Table 1:** Basline characteristics of the patients (N = 1048).

Characteristic	Uric acid (umol/L) (Tertile)			*p* value
	T1 (8.38–283.90)	T2 (284.60–389.30)	T3 (389.50–986.40)	
No. of patients	349	349	350	
Age (years, mean ± sd)	51.26 ± 11.90	50.11 ± 11.21	49.15 ± 11.22	0.052
Gender (female)	138 (39.54%)	73 (20.92%)	43 (12.29%)	< 0.001
BMI (Kg/m^2^, mean ± sd)	23.85 ± 4.11	25.24 ± 4.66	25.95 ± 4.55	< 0.001
Smoking	85 (24.36%)	92 (26.36%)	99 (28.29%)	0.499
Hypertension	234 (67.05%)	244 (69.91%)	248 (70.86%)	0.524
Diabetes	9 (2.58%)	8 (2.29%)	9 (2.57%)	0.962
Marfan syndrome	8 (2.29%)	15 (4.30%)	8 (2.29%)	0.195
Bicuspid aortic valve	5 (1.43%)	4 (1.15%)	2 (0.57%)	0.523
CRI	9 (2.58%)	2 (0.57%)	13 (3.71%)	0.019
Stroke	11 (3.15%)	13 (3.72%)	13 (3.71%)	0.896
Atherosclerosis	23 (6.59%)	18 (5.16%)	18 (5.14%)	0.635
Time to presentation (h, median (Q1–Q3))	36.00 (15.00–120.00)	24.00 (10.00–72.00)	18.00 (10.00–48.00)	0.777
SBP (mmHg, mean ± sd)	139.79 ± 28.37	139.98 ± 30.65	140.14 ± 33.05	0.988
DBP (mmHg, mean ± sd)	75.29 ± 18.33	75.77 ± 18.29	76.80 ± 20.07	0.557
Aortic diameter (mm)	44.55 ± 10.97	44.68 ± 10.48	44.10 ± 10.08	0.743
Aortic regurgitation	167 (47.85%)	155 (44.41%)	175 (50.00%)	0.329
Abdominal vessel involvement	131 (37.54%)	122 (34.96%)	128 (36.57%)	0.774
Arch vessel involvement	118 (33.81%)	111 (31.81%)	131 (37.43%)	0.284
EF value (%)	65.24 ± 7.01	64.82 ± 8.02	63.71 ± 9.26	0.037
Cr (umol/L median(Q1–Q3))	71.30 (54.10–86.30)	80.30 (65.50–110.00)	117.45 (83.23–164.88)	< 0.001
BUN (mmol/L median(Q1–Q3))	6.10 (4.54–8.27)	6.93 (5.39–9.06)	8.70 (6.36–13.06)	< 0.001
eGFR	94.30(72.37–124.04)	91.35(65.03–120.81)	65.51(42.56–103.19)	< 0.001
TG (mmol/L)	1.45 ± 0.86	1.54 ± 1.16	1.69 ± 1.35	0.019
TC (mmol/L)	3.89 ± 0.99	3.97 ± 0.94	4.00 ± 0.94	0.284
HDL (mmol/L)	1.45 ± 4.61	1.14 ± 0.31	1.33 ± 4.47	0.534
LDL (mmol/L)	2.02 ± 1.78	2.13 ± 0.85	2.24 ± 0.86	0.058
D-dimer (ug/mL)	4.00 (2.30–9.00)	3.79 (2.25–9.50)	4.53 (2.74–15.93)	0.019
FDP (ug/mL)	15.60 (7.60–35.30)	17.94 (7.49–51.29)	30.50 (11.65–67.58)	< 0.001
CRP (mg/L)	62.10 (13.10–116.00)	41.50 (9.72–108.00)	30.75 (8.23–89.38)	0.012
Operation	244 (69.91%)	232 (66.48%)	207 (59.14%)	0.009
Symptom				0.948
Chest pain	253 (72.49%)	248 (71.06%)	250 (71.43%)	
Back pain	45 (12.89%)	47 (13.47%)	44 (12.57%)	
Abdominal pain	12 (3.44%)	13 (3.72%)	13 (3.71%)	
Syncope	11 (3.15%)	18 (5.16%)	12 (3.43%)	
others	28 (8.03%)	23 (6.59%)	31(8.85%)	
Coronary malperfusion	8 (2.29%)	11 (3.15%)	40 (11.43%)	< 0.001
Mesenteric malperfusion	5 (1.43%)	4 (1.15%)	6 (1.71%)	0.819
Cerebral malperfusion	11 (3.15%)	18 (5.16%)	12 (3.43%)	0.334
Hypotension/shock	8 (2.29%)	16 (4.58%)	20 (5.71%)	0.071
Cardiac tamponade	2 (0.57%)	4 (1.15%)	8 (2.29%)	0.133
Mortality				< 0.001
Survivor	267 (76.50%)	238 (68.19%)	205 (58.57%)	
Non-survivor	82 (23.50%)	111 (31.81%)	145 (41.43%)	

*BMI* body mass index, *SBP* systolic blood pressure, *DBP* diastole blood pressure, *Cr* creatinine, *BUN* blood urea nitrogen, *eGFR* estimated glomerular filtration rate, *TG* triglyceride, *TC* total cholesterol, *HDL* high-density lipoprotein, *LDL* low-density lipoprotein, *FDP* fibrinogen and fibrin degradation products, *CRP* C-reactive protein, *CRI* chronic renal insufficiency, *EF* ejection fraction.

### Univariate analysis

Table 2 displays the univariate analyses, which revealed that gender, BMI, diabetes, smoking status, Marfan syndrome, hypertension, bicuspid aortic valve, CRI, time to presentation, aortic diameter, aortic regurgitation, abdominal vessel involvement, arch vessel involvement, mesenteric malperfusion, cerebral malperfusion, cardiac tamponade, EF value, and LDL did not contribute to the outcome variable. However, the results showed that age, stroke, atherosclerosis, coronary malperfusion, hypotension/shock, back pain, Cr, BUN, TG, TC, and UA presented positive correlation with the outcome variable, whilst SBP, DBP, HDL, and operation status depicted negative correlation with the outcome variable.

**Table 2:** Univariate analysis for in-hospital mortality.

	Statistics	OR (95%CI)	*p* value
Age (years)	50.17 ± 11.47	1.03 (1.01, 1.04)	< 0.001
Gender (female)	254 (24.24%)	0.89 (0.65, 1.21)	0.448
BMI (Kg/m^2^)	25.01 ± 4.53	1.02 (0.99, 1.05)	0.197
Smoking	276 (26.34%)	0.74 (0.55, 1.00)	0.051
Hypertension	726 (69.27%)	1.31 (0.98, 1.74)	0.066
Diabetes	26 (2.48%)	1.56 (0.71, 3.43)	0.270
Marfan syndrome	31 (2.96%)	0.86 (0.39, 1.88)	0.697
Bicuspid aortic valve	11 (1.05%)	1.20 (0.35, 4.14)	0.770
CRI	24 (2.29%)	2.14 (0.95, 4.82)	0.066
Stroke	37 (3.53%)	3.23 (1.65, 6.30)	0.001
Atherosclerosis	59 (5.63%)	2.29 (1.35, 3.88)	0.002
Time to presentation (h, median(Q1–Q3))	24.00 (11.00–72.00)	1.00 (1.00, 1.00)	0.427
SBP (mmHg)	139.97 ± 30.72	0.99 (0.99, 1.00)	0.002
DBP (mmHg)	75.95 ± 18.91	0.99 (0.98, 1.00)	0.002
Aortic diameter (mm)	44.45 ± 10.51	1.01 (1.00, 1.02)	0.213
Aortic regurgitation	497 (47.42%)	0.88 (0.68, 1.14)	0.335
Abdominal vessel involvement	381 (36.35%)	0.93 (0.71, 1.22)	0.594
Arch vessel involvement	497 (47.42%)	0.88 (0.68, 1.14)	0.335
EF value (%)	65.07 ± 8.14	0.99 (0.97, 1.01)	0.345
Cr (umol/L)	113.86 ± 121.30	1.00 (1.00, 1.00)	0.002
BUN (mmol/L)	12.56 ± 21.70	1.01 (1.00, 1.01)	0.041
eGFR	126.95 ± 200.76	1.00 (1.00, 1.00)	0.110
TG (mmol/L)	1.56 ± 1.14	1.16 (1.03, 1.31)	0.012
TC (mmol/L)	3.95 ± 0.96	1.15 (1.01, 1.32)	0.040
HDL(mmol/L)	1.31 ± 3.71	0.50 (0.32, 0.76)	0.001
LDL (mmol/L)	2.13 ± 1.24	0.93 (0.81, 1.06)	0.293
D-dimer (ug/mL)	9.61 ± 11.78	1.03 (1.02, 1.04)	< 0.001
FDP (ug/mL)	44.29 ± 66.84	1.00 (1.00, 1.01)	< 0.001
CRP (mg/L)	66.67 ± 68.05	1.00 (0.99, 1.00)	< 0.001
Operation	683 (65.17%)	0.04 (0.03, 0.06)	< 0.001
Symptom			
Chest pain	751 (71.66%)	Ref	
Back pain	136 (12.98%)	1.53 (1.05, 2.23)	0.027
Abdominal pain	38 (3.63%)	0.60 (0.27, 1.33)	0.208
Syncope	41 (3.91%)	1.44 (0.75, 2.75)	0.268
Others	82 (7.82%)	1.13 (0.67, 1.90)	0.658
Coronary malperfusion	59 (5.63%)	2.29 (1.35, 3.88)	0.002
Mesenteric malperfusion	15 (1.43%)	0.76 (0.24, 2.41)	0.642
Cerebral malperfusion	41 (3.91%)	1.36 (0.72, 2.59)	0.346
Hypotension/shock	44 (4.20%)	2.18 (1.19, 3.99)	0.012
Cardiac tamponade	14 (1.34%)	2.12 (0.74, 6.10)	0.162
UA(umol/L, per 10 increments)	34.65 ± 13.54	1.03 (1.02, 1.04)	< 0.001

*BMI* body mass index, *SBP* systolic blood pressure, *DBP* diastole blood pressure, *Cr* creatinine, *BUN* blood urea nitrogen, *eGFR* estimated glomerular filtration rate, *TG* triglyceride, *TC* total cholesterol, *HDL* high-density lipoprotein, *LDL* low-density lipoprotein, *FDP* fibrinogen and fibrin degradation products, *CRP* C-reactive protein, *CRI* chronic renal insufficiency, *EF* ejection fraction.

### Unadjusted and adjusted model results

Following adjustment for potential confounding factors, the impact of serum UA on in-hospital mortality was deduced based on three models. Table 3 presents the corresponding effect values (OR) and 95% confidence intervals. The adjusted covariates, with the exception of symptom, coronary malperfusion, mesenteric malperfusion, cerebral malperfusion, hypotension/shock, cardiac tamponade, and operation, for the non-adjusted model and model I are shown in Table 1; with every 10 µmol/L increase in admission serum UA, in-hospital mortality showed a 3% increase, with OR and 95% confidence intervals of (1.03, 95% CI 1.02–1.04) and (1.03, 95% CI 1.02–1.05), respectively. Model II represents the fully adjusted version of model I, including symptom, coronary malperfusion, mesenteric malperfusion, cerebral malperfusion, hypotension/shock, cardiac tamponade and operation, whereby each additional 10 µmol/L increase in admission serum UA resulted in an increase of in-hospital mortality by 4% (1.04, 95% CI 1.02–1.06). Focusing on the adjusted model, the p value pertaining to the trend of admission serum UA with categorical variables was found to be consistent with the outcome when admission serum UA was indicated as a constant variable following the conversion of UA from a continuous to categorical variable (tertile). Nonetheless, when the admission serum UA was presented as a categorical variable in the fully adjusted model, the effective value trend in the alternative UA group was found to be non-equidistant. This finding suggests the presence of a nonlinear relationship between admission serum UA and in-hospital mortality.

**Table 3:** Relationship between Uric acid and in-hospital mortality in different models.

Exposure	Crude model (OR, 95%CI, *p*)	Model I (OR, 95%CI, *p*)	Model II (OR, 95%CI, *p*)
UA (umol/L, per 10 increments)	1.03 (1.02, 1.04) < 0.001	1.03 (1.02, 1.05) < 0.001	1.04 (1.02, 1.06) < 0.001
**UA (umol/L) (tertile)**			
T1	Ref	Ref	Ref
T2	1.52 (1.09, 2.12) 0.014	1.50 (1.02, 2.22) 0.042	1.76 (1.05, 2.95) 0.031
T3	2.30 (1.66, 3.19) < 0.001	2.38 (1.57, 3.59) < 0.001	2.77 (1.60, 4.79) < 0.001
*p* for trend	< 0.001	< 0.001	< 0.001

Crude Model adjusted for none.

Model I adjusted for age, gender, BMI, smoking, hypertension, diabetes, Marfan syndrome, Bicuspid aortic valve, CRI, stroke, atherosclerosis, time to presentation, SBP, DBP, aortic diameter, aortic regurgitation, abdominal vessel involvement, arch vessel involvement, EF value, Cr, BUN, eGFR, TG, TC, HDL, LDL, D-dimer, FDP, CRP.

Model II adjusted for Model I and symptom, coronary malperfusion, mesenteric malperfusion, cerebral malperfusion, hypotension/shock, cardiac tamponade and operation.

*BMI* body mass index, *SBP* systolic blood pressure, *DBP* diastole blood pressure, *Cr* creatinine, *BUN* blood urea nitrogen, *eGFR* estimated glomerular filtration rate, *UA* uric acid, *TG* triglyceride, *TC* total cholesterol, *HDL* high-density lipoprotein, *LDL* low-density lipoprotein, *FDP* fibrinogen and fibrin degradation products, *CRP* C-reactive protein, *CRI* chronic renal insufficiency, *EF* ejection fraction.

### Nonlinearity results between admission serum UA and in-hospital mortality

**Figure 2:**
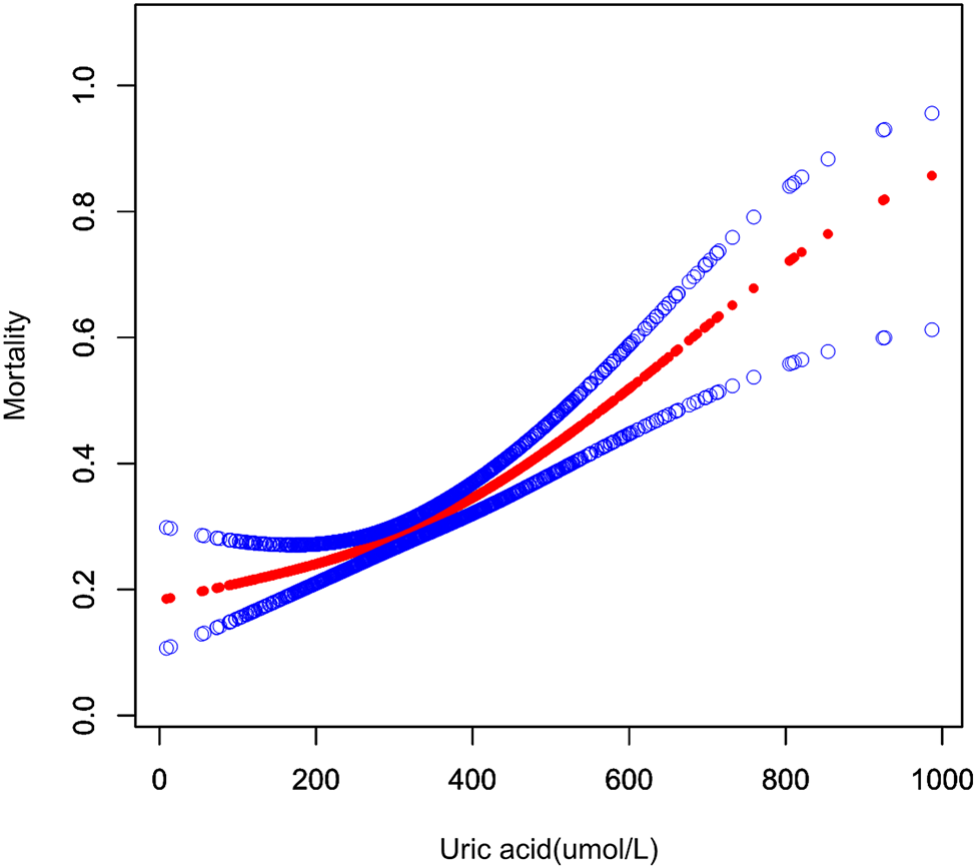
Relationship between serum uric acid and in-hospital mortality; this was non-linear (*p* < 0.001) in a generalised additive model (GAM). The smooth curve fit between variables is represented by the red line, whilst the 95% confidence interval from the fit is shown as blue bands. Adjustments have been made for patient age, gender, smoking status, BMI, diabetes, hypertension, Marfan syndrome, bicuspid aortic valve, CRI, atherosclerosis, stroke, time to presentation, aortic diameter, aortic regurgitation, arch vessel involvement, abdominal vessel involvement, EF value, SBP, DBP, Cr, BUN, eGFR, TG, TC, HDL, LDL, D-dimer, FDP, CRP, symptom, coronary malperfusion, mesenteric malperfusion, cerebral malperfusion, hypotension/shock, cardiac tamponade and operation status.

Non-linear correlation between admission serum UA and in-hospital mortality was determined based on the smooth curve, following adjustment for covariates (Table 4, Fig. 2). The linear regression model and two-piecewise linear regression model were used respectively to achieve this finding; *p* < 0.05 for the log-likelihood ratio test. Resultantly, dual piecewise linear regression was deemed to be the most suitable approach to deduce the potential association between admission serum UA and in-hospital death. Based on the results of recursive algorithm and two-piecewise linear regression, the premeditated inflection point was 260 µmol/L. When UA ≤ 260 µmol/L, the effect size and 95% CI were 1.00 and 0.99–1.02, respectively (UA per 10 increments). When UA > 260 µmol/L, the effect size and 95% CI were 1.04 and 1.02–1.05, respectively (UA per 10 increments).

**Table 4:** The results of the two-piecewise linear model (UA per 10 increments).

	Mortality (OR, 95%CI)	*p* value
Fitting model by standard linear regression	1.04 (1.02, 1.06)	< 0.001
Fitting model by two-piecewise linear regression		
Inflection point of UA (umol/L)	260	
≤ 260	1.00 (0.99, 1.02)	0.419
> 260	1.04 (1.02, 1.05)	< 0.001
*p* for log-likelihood ratio test	0.030	

Adjusted: age, gender, BMI, smoking, hypertension, diabetes, Marfan syndrome, Bicuspid aortic valve, CRI, stroke, atherosclerosis, time to presentation, SBP, DBP, aortic diameter, aortic regurgitation, abdominal vessel involvement, arch vessel involvement, EF value, Cr, BUN, TG, TC, HDL, LDL, symptom, coronary malperfusion, mesenteric malperfusion, cerebral malperfusion, hypotension/shock, cardiac tamponade and operation.

*BMI* body mass index, *SBP* systolic blood pressure, *DBP* diastole blood pressure, *Cr* creatinine, *BUN* blood urea nitrogen, *eGFR* estimated glomerular filtration rate, *UA* uric acid, *TG* triglyceride, *TC* total cholesterol, *HDL* high-density lipoprotein, *LDL* low-density lipoprotein, *FDP* fibrinogen and fibrin degradation products, *CRP* C-reactive protein, *CRI* chronic renal insufficiency, *EF* ejection fraction.

### Subgroup analysis

Data pertaining to participant gender, age, BMI, smoking status, diabetes, hypertension, CRI, eGFR, D-dimer, FDP, CRP, abdominal vessel involvement, coronary malperfusion, mesenteric malperfusion, cerebral malperfusion, hypotension/shock, cardiac tamponade and operation status represented the stratification variables that were used to ascertain the corresponding development of effect sizes (Table 5). Participant subgroup analysis indicated a stable relation between UA and in-hospital mortality, whilst statistical significance was undetected between the subgroups.

**Table 5:** Results of subgroup analysis and interaction analysis (UA per 10 increments).

Characteristic	No	OR	95%CI Low	95%CI High	*p* (interaction)
**Age(years)**					0.077
< 60	815	1.04	1.03	1.05	
≥ 60	233	1.01	0.99	1.03	
**Gender**					0.610
Male	794	1.03	1.02	1.04	
Female	254	1.03	1.01	1.06	
**BMI (Kg/m**^**2**^**)**					0.652
< 18.5	47	1.02	0.97	1.07	
≥ 18.5, < 23	315	1.03	1.01	1.05	
≥ 23	686	1.03	1.02	1.04	
**Smoking**					0.451
No	772	1.03	1.02	1.04	
Yes	276	1.03	1.00	1.05	
**Hypertension**					0.142
No	322	1.04	1.02	1.06	
Yes	726	1.03	1.02	1.04	
**Diabetes**					0.959
No	1022	1.03	1.02	1.04	
Yes	26	1.02	0.97	1.08	
**CRI**					0.621
No	1024	1.03	1.02	1.04	
Yes	24	1.03	0.99	1.07	
**eGFR**					0.575
< 120	798	1.03	1.02	1.04	
≥ 120	250	1.02	1.00	1.05	
**D-dimer (ug/mL)**					0.109
Low (0.01–4.09)	523	1.03	1.01	1.04	
High (4.10–40.00)	525	1.04	1.02	1.05	
**FDP (ug/mL)**					0.121
Low (0.60–20.10)	523	1.02	1.00	1.03	
High (20.12–473.62)	525	1.03	1.02	1.05	
**CRP (mg/L)**					0.808
Low (0.13–42.40)	523	1.03	1.01	1.04	
High (42.70–368.00)	525	1.03	1.02	1.05	
**Abdominal vessel involvement**					0.656
No	667	1.03	1.02	1.04	
Yes	381	1.03	1.02	1.05	
**Coronary malperfusion**					0.083
No	989	1.03	1.01	1.04	
Yes	59	1.09	1.03	1.14	
**Mesenteric malperfusion**					0.945
No	1033	1.03	1.02	1.04	
Yes	15	1.03	0.95	1.13	
**Cerebral malperfusion**					0.989
No	1007	1.03	1.02	1.04	
Yes	41	1.03	0.99	1.08	
**Hypotension/shock**					0.614
No	1004	1.03	1.02	1.04	
Yes	44	1.02	0.98	1.06	
**Cardiac tamponade**					0.077
No	1034	1.03	1.02	1.04	
Yes	14	1.19	0.95	1.50	
**Operation**					0.337
No	365	1.03	1.01	1.04	
Yes	683	1.04	1.02	1.06	

*BMI* body mass index, *CRI* chronic renal insufficiency, *eGFR* estimated glomerular filtration rate, *FDP* fibrinogen and fibrin degradation products, *CRP* C-reactive protein.

### Survival curve analysis

**Figure 3:**
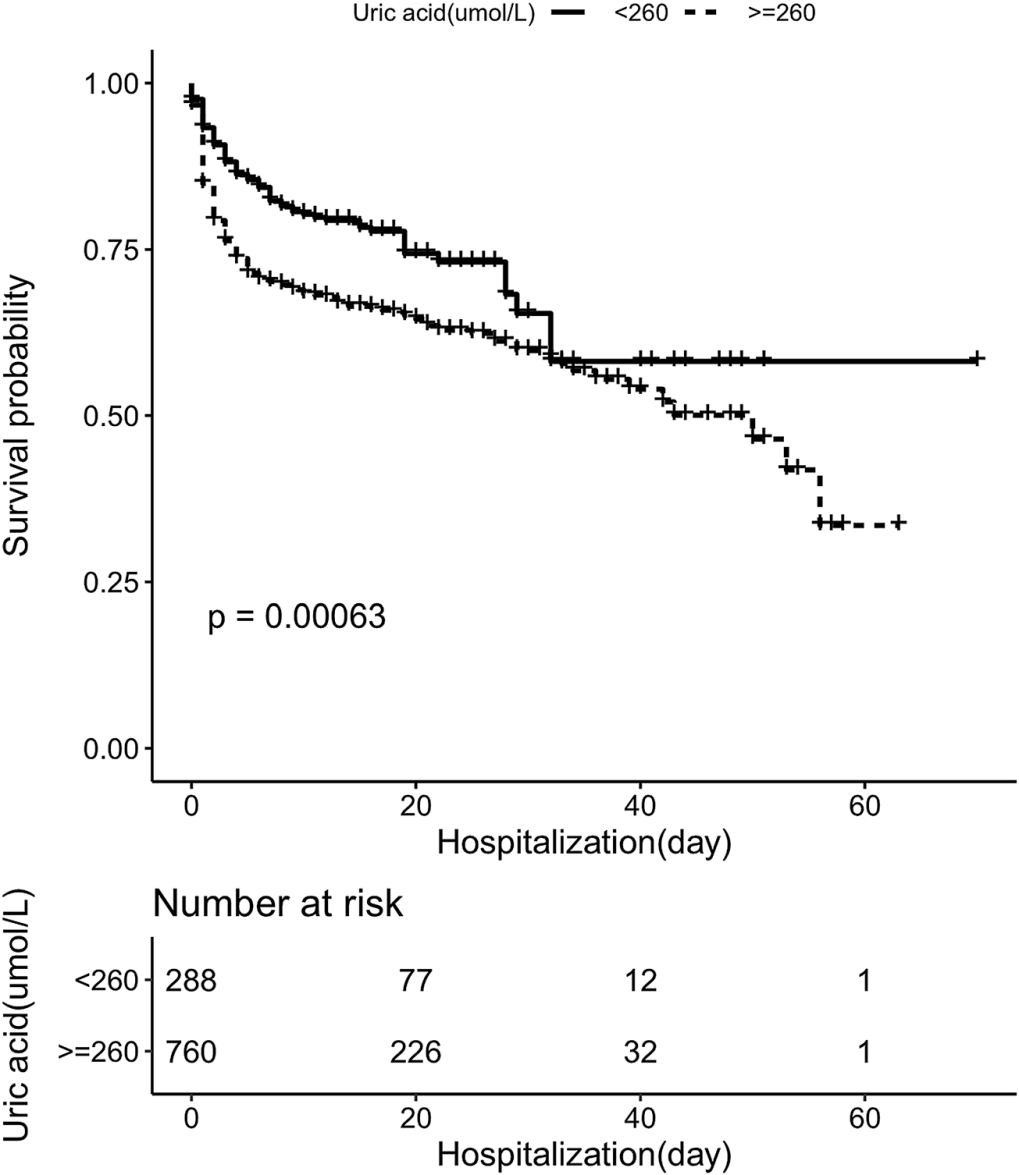
In-hospital survival curve analysis based on patients with ATAAD.

The results of Kaplan–Meier analysis demonstrate a significantly higher in-hospital survival rate in patients with admission serum UA level ≤ 260 µmol/L (*p* < 0.05) (Fig. 3).

## Discussion

In the fully adjusted model, admission serum UA showed positive correlation with in-hospital mortality when admission serum UA > 260 µmol/L: an increase of 10 µmol/L in admission serum UA was linked to a 4% increase in in-hospital mortality, according to the model-based effect sizes. However, when admission serum UA ≤ 260 µmol/L, this relationship was not detected [1.00 (95%CI 0.99–1.02), *p* = 0.419]. Furthermore, nonlinearity was established between admission serum UA and in-hospital mortality.

Despite these findings, the mechanism behind this correlation remains unclear. Previous research has determined that UA plays a contributory role in the proliferation and vasoconstriction of vascular smooth muscle cells^16,17^. In addition, UA activates the intrarenal renin-angiotensin system and enhances angiotensin II expression in vascular endothelial cells^18,19^. UA has also been shown to facilitate the stimulation of human mononuclear cells and subsequent production of IL-1ß, IL-6, and TNF-α; this has been linked to the generation of monocyte chemoattractant protein-1 (MCP-1)^20^, which contributes to the initiation of aortic dissection^21^. Moreover, increased UA levels have been suggested to exacerbate the production of reactive oxygen species, thus enhancing oxidative stress, and leading to aortic media lesions^14,22^. A further consequence of increased UA is damage to the vascular structure through enhanced inflammation, thereby weakening the aortic wall^23,24^. However, insufficient evidence is currently available to determine whether hyperuricemia treatment could reduce the risk of mortality due to ATAAD.

According to previous research, Lapsia et al.^25^ retrospectively analyzed 190 patients undergoing cardiovascular surgery to study serum uric acid levels and the risk of AKI after surgery, and found that patients with elevated preoperative serum uric acid levels had a significantly increased risk of AKI after surgery. In a subgroup analysis (Table 5), we found that UA was associated with ATAAD prognosis in the operation group with a 4% increased risk of death per 10 µmol/L increase in UA and the 95% CI was 1.02–1.06. One of the possible reasons for this may be the higher the preoperative UA levels, the higher the risk of AKI after surgery. In addition, UA was closely related to renal function, and hyperuricemia was likely to occur when renal impairment^26^. Circulatory disorders of the kidney caused by the aortic dissection may cause acute renal injury, and renal function is traditionally measured using eGFR during preoperative risk assessment^27^. Therefore, in this study, we collected preoperative eGFR levels in patients with ATAAD and found that the relationship between UA and ATAAD prognosis was stable in different eGFR group (Table 5).

Research by Otaki et al.^28^ indicated that hyperuricemia had a higher mortality rate linked to AD in the general population, and thus, is an independent risk factor in this context. Also, the incidence of AD-related death increased in a linear pattern as UA levels increased. However, data was not attained regarding the type of aortic dissection or the therapies used, such as surgical and endovascular aortic repair; these are significant factors as they have been associated with aortic dissection prognosis. In another study focusing on patients with ATAAD, Zhang et al.^29^ found that increased admission serum UA level can independently predict in-hospital mortality (OR = 1.010, 95% CI 1.005–1.015, *p* < 0.001). Yet it should be acknowledged that nonlinearity was not performed in this study, and a relatively small sample size of patients with ATAAD was included. The present study findings resulted in the construction of a J-shaped curve and threshold effect in regard to the relationship between admission serum UA and in-hospital mortality in this specific patient population.

These findings are believed to be the first to observe the threshold effect in the context of admission of in-hospital mortality and UA in patients with ATAAD. Furthermore, this study is expected to be a valuable reference point for prospective research regarding the formation of predictive and diagnostic models of in-hospital death rates in patients with ATAAD.

An evaluation of the study presents strengths such as the opportunity to explore this area on a deeper level due to the nonlinearity outcome; in addition, the observational nature of the study means that there is minimal risk to participants. Also, calculated adjustments were implemented to alleviate residual confounders. Another strength of the study is that the independent variables were treated in the same way as the continuous and categorical variables, thereby reducing contingency in the subsequent data analysis and enhancing the validity of the study outcomes. Finally, the quality of the data was improved as a result of the effect modifier factor analysis, thus enabling the generation of a steadfast conclusion regarding the diverse subgroups.

Despite these positive aspects, certain limitations of the study were also identified. The main drawback is that the participants were all Chinese, and thus, the findings cannot be generalised on a universal scale relating to other ethnicities. Another limitation is that anti-dyslipidemia, anti-hypertensive, anti-diabetic drugs, and anti-hyperuricemic drugs have been reported to affect serum UA level. However, data regarding the medications taken by the participants in this study was not available; consequently, the participants’ serum UA levels may have been influenced by certain medications, which would have skewed the study findings.

## Conclusions

Overall, it can be deduced that a non-linear relationship exists between admission serum UA and in-hospital mortality. Moreover, correlation between admission serum UA and in-hospital mortality is positive when serum UA exceeds 260 µmol/L.

## Data Availability

The datasets used and/or analysed during the current study available from the corresponding author on reasonable request.
